# Importance of Visual Estimation of Coronary Artery Stenoses and Use of Functional Evaluation for Appropriate Guidance of Coronary Revascularization—Multiple Operator Evaluation

**DOI:** 10.3390/diagnostics11122241

**Published:** 2021-11-30

**Authors:** Lucian Calmac, Nicoleta-Monica Popa-Fotea, Vlad Bataila, Vlad Ploscaru, Adrian Turea, Irina Andra Tache, Diana Stoian, Lucian Itu, Elisabeta Badila, Alexandru Scafa-Udriste, Maria Dorobantu

**Affiliations:** 1 Department of Cardiology, Emergency Clinical Hospital, 8 Calea Floreasca, 014461 Bucharest, Romania; vladbataila@yahoo.co.uk (V.B.); vlad_ploscaru86@yahoo.com (V.P.); elisabeta.badila@umfcd.ro (E.B.); alexscafa@yahoo.com (A.S.-U.); 2 Department Cardio-Thoracic, University of Medicine and Pharmacy “Carol Davila”, 8 Eroii Sanitari, 050474 Bucharest, Romania; maria.dorobantu@gmail.com; 3 Department of Image Fusion and Analytics, Siemens SRL, 78 B-dul 15 Noiembrie, 5000978 Brasov, Romania; adrian.turea@siemens.com (A.T.); irina.tache.ext@siemens.com (I.A.T.); diana.stoian@siemens.com (D.S.); lucian.itu@siemens.com (L.I.); 4 Department of Automation, Polytechnic University of Bucharest, 313 Splaiul Independenței, 060042 Bucharest, Romania; 5 Department of Automation and Applied Informatics, Transilvania University of Brasov, 5 Mihai Viteazul, 500174 Brasov, Romania

**Keywords:** fractional flow reserve, visual estimation, agreement, diameter stenosis, coronary artery disease, functional evaluation, inter-observer variability, group decision, myocardial ischemia, cardiac imaging

## Abstract

Background: Visual estimation (VE) of coronary stenoses is the first step during invasive coronary angiography. The aim of this study was to evaluate the accuracy of VE together with invasive functional assessment (IFA) in defining the functional significance (FS) of coronary stenoses based on the opinion of multiple operators. Methods: Fourteen independent operators visually evaluated 133 coronary lesions which had a previous FFR measurement, indicating the degree of stenosis (DS), FS and IFA intention. We determined the accuracy of FS prediction using several scenarios combining individual and group decision, considering IFA as deemed necessary by the operator or only in intermediate lesions. Results: The accuracy of VE in predicting FS was largely variable between operators (average 66.1%); it improved significantly when IFA was used either as per operator’s opinion (86.3%; *p* < 0.0001) or only in intermediate DS (82.9; *p* < 0.0001). There was no significant difference between using IFA per observer’s opinion or only in intermediate DS lesions (*p* = 0.166). The poorest accuracy of VE for FS was obtained in intermediate DS lesions (59.1%). Conclusions: There are significant inter-observer differences in reporting the degree of DS, while the accuracy of VE prediction of FS is also largely dependent on the operator, and the worst performance is obtained in the evaluation of intermediate DS.

## 1. Introduction

Visual evaluation of coronary stenoses angiographic appearance (also known as eyeballing) by the interventional cardiologist represents the first evaluation step during invasive coronary angiography (ICA). It informs the physician about need for further anatomical or functional evaluation (either invasive or non-invasive) or supports the therapeutic decision regarding the need for revascularization. One of the key issues during stenosis evaluation is the quantification of the degree of stenosis. This may be performed either visually (visual estimation–VE) or using dedicated software packages for quantitative coronary angiography (QCA) to estimate the degree of diameter stenosis (DS). The percentage DS (%DS) is calculated by reporting the minimum diameter of stenosis to the reference diameter. There are two current methods for determining the %DS: the first one averages the proximal and distal reference vessel diameter and the second one interpolates the reference vessel diameter [1]. According to current guidelines, there are different DS thresholds which are recommended to guide the indication for coronary revascularization. According to the %DS, lesions may be classified as probably nonsignificant functionally (DS < 50% in the European and <40% in the American guideline) or probably functional significant (DS > 90% in the European and >70% in the American guideline). The effect of a 50% DS on maximum achievable coronary flow was firstly formulated by Gould in 1974 on an experimental model of coronary stenosis [2]. Subsequently, more complex elements were proposed to comprehensively evaluate the severity of coronary plaques, such as eccentricity of the plaques, bifurcation lesions, morphological and multidimensional geometric parameters derived from computer tomography imaging [3]. Moreover, with angiography we evaluate the % DS based on a 2D projection of a 3D vessel structure which may not correctly reflect the area reduction induced by the stenosis, the latter being considered a more reliable parameter for the evaluation of the functional impact of an atherosclerotic lesion [4].

The use of coronary physiology to guide the decision of revascularization has been proven to improve long term patient outcomes compared to angiographic guidance [5]. The fractional flow reserve (FFR), calculated as the ratio of the pressure measured distally to a stenosis to the proximally measured pressure during maximal hyperemia, can determine objectively the functional significance of coronary stenosis using a threshold of 0.8 [6]. This is most relevant in the evaluation of intermediate lesions, defined as 50–90% DS (European guidelines) or 40–70% DS (American guidelines) when there is no proof for ischemia prior of coronary angiography. These DS thresholds rely however mainly on VE. Significant mismatch between visual and functional severity of DS have been observed and pinpointed in earlier studies [7,8]. Seen these disparities, the VE of %DS on ICA is being challenged as the gold standard for the hemodynamic significance of a lesion, being replaced with FFR [9].

In patients with ICA, prior documentation of ischemia is present in only 44% of cases [10], but the percentage largely depends on current practices and test availability [11]. For patients with multivessel disease (defined as more than one major coronary artery with DS > 50%), even if prior proof of ischemia is obtained, it is important to define the functional significance of each stenosis for a proper guidance of coronary revascularization. Limitations of a more extensive use of invasive functional evaluation at the time of ICA include costs, increased procedural time [12] or reduced experience of the operator [13].

Based on real-world observational data, a recent report [10] shows that in patients with stable coronary disease and angiographic intermediate lesions (40–70% DS based on VE), FFR was used in only 16.5% of cases, while prior noninvasive tests were performed just in 38.7% of patients. The same report shows that less than one third of the patients which were revascularized (29.5%) without invasive functional evaluation had a prior noninvasive proof of ischemia. Hence, it is obvious that VE plays an important role in predicting the functional significance and the need for subsequent revascularization. Zir et al. [14] established 45 years ago the limitations in VE accuracy, documenting a significant inter-observer variability in DS assessment. That is why we sought to evaluate the variability in visual interpretation by practicing interventional cardiologists, in the current era when there is strong evidence about the need to pursue a functional strategy for coronary revascularization guidance.

The main aim of the present study is to evaluate the accuracy of VE in establishing the need for revascularization based on the evaluation of multiple operators, compared to invasive FFR with threshold of 0.8 as gold standard. Also, we evaluated the inter-operator variability of VE in grading the severity of coronary stenoses (degree of DS). Furthermore, we sought to identify individual predictors for the decision to perform an invasive functional evaluation for establishing the functional significance (stenosis severity, lesion location, etc.), as well as to assess the performance of the selective use of FFR (based either on operator opinion after VE or degree of DS as evaluated visually).

## 2. Material and Methods

Invasive coronary angiography evaluations were performed in 86 patients as per local practice based on clinical indication for evaluation, between 2014 and 2016, for the purpose of a local prospective study aiming at the hemodynamic characterization of coronary lesions in relation to angiographic appearance. Briefly, this cohort comprised patients needing invasive functional evaluation for at least one lesion based on the clinical judgement of the attending physician after ICA. The exclusion criteria were the following: subjects with acute coronary syndromes during previous 1 week, occluded of sub-totally occluded vessels, left main lesions or previous myocardial infarction in the investigated vessel. The protocol was approved by the local ethical committee and all patients provided informed consent prior to the inclusion. Investigators were advised to perform at least two projections for each coronary segment separated by at least 30 degrees in rotation/angulation, to allow for an accurate visualization of the coronary stenosis. After standard image acquisition, the physician performed FFR measurements in all coronary segments which had a clinical indication, using intracoronary administration of adenosine as per general recommendations for FFR measurement. Coronary revascularization was decided by the attending physician based on the clinical and invasive evaluation (including the FFR measurements), using clinical practice guidelines recommendations.

The images were anonymized, and two different views showing worst angiographic stenosis were selected for each lesion for which FFR was available. These views were included in a web-based annotation application which was developed for the purpose of this study. Each pair of selected images was evaluated independently by certified interventional cardiologists (which are regularly involved in taking decisions regarding coronary revascularization) who accepted to take part in the study. The evaluation process was performed between January and March 2021. Because this process was based entirely on anonymized images, no further ethical committee approval was deemed necessary.

For each lesion the operators were asked to answer to the following:

- Indicate the segment of the most severe lesion on each major coronary artery (LAD, left circumflex (LCx) or right coronary artery (RCA)).
- Quantify the most severe degree of DS by choosing one of the following intervals: <30%; 30–50%; 50–60%; 60–70%; 70–80%; 80–90%; >90%. During data analysis several other DS severity intervals were considered by concatenating the initial intervals (<50%; 50–70%; ≥70%).
- Indicate whether the detected lesion should be revascularized based on the operator opinion, in accordance with his/her regular practice.
- Indicate whether the operator would need invasive functional evaluation to establish the indication for revascularization, according to his/her regular practice.

During data evaluation we designed several scenarios:

- Decision of each operator based only on VE.
- Decision of majority–the decision which gathered at least eight positive votes (out of 14 votes) for functional evaluation, or for the need for revascularization.
- Decision of the top three operators–the decision which gathered at least 3 votes from the best three operators (selected based on the overall accuracy in predicting the functional significance of the lesions (FFR ≤ 0.8).
- Hybrid 1–used the operator decision for revascularization for the lesions where functional invasive evaluation was not deemed necessary, while the decision based on the result from FFR measurement was used when it was required by the operator.
- Hybrid 2–used the operator decision for revascularization for non-intermediate lesions (<50% and ≥70%), while the decision based on the result from FFR measurement was used for the lesions graded as being intermediate (degree of DS 50–70%).

The performance of visual DS grading was assessed initially using the 7-degree scale (see above), for general agreement between all operators. For each lesion we determined also the DS determined based on the vote of the majority. For further analyses we reclassified the visual DS based on a three-grade scale using the 50% and 70 % thresholds, and this classification was again analyzed for inter-observer agreement. We also used this 3-grade classification as a nominal variable because of the different clinical significance for each class: <50%–probably non-significant lesions; 50–70%–intermediate lesions; >70%–probably significant lesions.

### Statistical Analysis

All analyses were conducted using the statistical software program SPSS version 23 (IBM, Armonk, NY, USA). Data was presented as mean ± SD for continuous variables and as number and percentage for categorical variables. Contingency tables were generated for each of the 14 operators to assess the agreement between the indication for revascularization as per his/her VE and FFR as proof of ischemia (using ≤0.8 as threshold). Kendall’s W was employed to determine the agreement between the operators’ VE concerning the severity of each lesion evaluated based on a seven-or three-degree severity scale; a Kendall W value of one depicting perfect agreement and zero, no agreement. The agreement concerning revascularization or the necessity for functional evaluation between two observers was calculated by Cohen’s kappa, and between multiple observers by Fleiss kappa. We also used Fleiss kappa for the inter-observer agreement regarding 3-grade DS lesion classification when we interpreted the categories on the clinical significance basis (<50%–probably non-significant; 50–70%–indeterminate; >70% probably significant). The kappa agreement was interpreted as follows: poor (<0.1), slight (0.1–0.2), fair (0.21–0.4), moderate (0.41–0.6), substantial (0.61–0.8), almost perfect (0.81–1), or perfect (1). For each observer sensitivity, specificity, the positive (PPV) and negative predictive values (NPV) and the accuracy (ACC) were calculated. For comparing the various scenarios of revascularization (VE, hybrid 1, hybrid 2) we used the McNemar test. The Phi correlation coefficient was calculated to evaluate the correlation between the decision to perform invasive testing and several variables, such as the localization of the stenosis (proximal or distal), the artery (LAD, LCx, RCA) or the visual grading. The proximal segments of major coronary arteries were considered as follows: 1 (RCA), 11 (LCx) and 6 (LAD). The receiver operator characteristic (ROC) analysis was used to evaluate the diagnostic capability of the VE of DS to detect hemodynamically significant stenoses (based on a FFR value ≤ 0.80).

## 3. Results

Complete evaluation of all lesions was performed by 14 interventional cardiologists. Based on their reporting they had a previous experience in interventional cardiology of 12.9 ± 6.7 (5–26) years, with an annual PCI load of 234 ± 124.8 (50–400) procedures. The cohort included 86 patients with stable coronary syndrome, with a total number of 133 lesions. In our study, 67.4% of patients were male with a mean age of 62.6 ± 8.9 years. A percentage of 88.3 of the patients were hypertensive, 30.2% were diabetic and 51.16% were smokers (Table 1). Most of the evaluated lesions were located on the LAD (71 lesions, 53.38%), whereas 32 were on RCA (24.06%), and 30 (22.5%) on LCx. Among all evaluated lesions, 39.09% (52 lesions) were hemodynamically significant when assessed by FFR with a mean FFR value of 0.67 (range 0.19–0.8), while the mean FFR was 0.88 (0.81–0.98) in lesions with non-ischemic FFR. Subsequent revascularization was performed by PCI in 44 subjects (51 lesions), while in four patients (five lesions) the decision was towards surgical revascularization. The general and FFR related characteristics are displayed in Table 1.

The results for the VE of the stenoses severity are presented in Table A1 (Appendix A). The agreement for the VE of lesions among all observers was good both on the 7-degree scale (Kendall’s W = 0.866, *p* < 0.005) and on the 3-degree scale (Kendall’s W = 0.748, *p* < 0.005). Based on the classification of the majority, 25 (18.8%) lesions had DS < 50%, 60 (45.1%) had DS 50–70% and 48 (36.1%) had DS > 70%. For all operators the individual gradings for the 3-degree scale are presented in Table A2 and Table A3. For each interval of DS there were 5–57, 29–90 and 16–83 lesions classified as <50%, 50–70% and respectively >70%. When the 3-gade scale agreement was measured using Fleiss kappa for nominal variables, there was an overall fair agreement (k = 0.392 ± 0.07; *p* < 0.0001) and a moderate agreement between the decision of the top three operators (k = 0.442 ± 0.037; *p* < 0.0001).

Invasive functional evaluation was variably requested by different operators in 27.3–74.6% of the cases (mean value of 49.2%) (Table A4). The agreement for the necessity to proceed to invasive-functional tests among all operators was only fair, with a Fleiss kappa of 0.214. For lesions evaluated as <50%, 50–70% and >70% the functional evaluation was considered necessary in 0–76,9%, 60–100% and 0–67.7% respectively, by different operators resulting in mean values of 22.8%, 88.3% and 29% for each category. It was not possible to evaluate the agreement based on the degree of DS because the lesions were graded differently by different evaluators. For the top three operators there was only a moderate overall agreement, (average Cohen kappa 0.534) (Table 2). However, when comparing the decision of the majority (at least eight positive votes) to the majority of the top three operators, the agreement was much better (Cohen k 0.729). The decision to perform functional assessment was not influenced by the type of vessel (LAD, LCx or RCA) or the localization of the lesion (proximal versus distal) but correlated for the majority of the investigators (11 of them) with visual classification as intermediate stenosis (phi value 0.204–0.763–Table 3).

**Figure 1:**
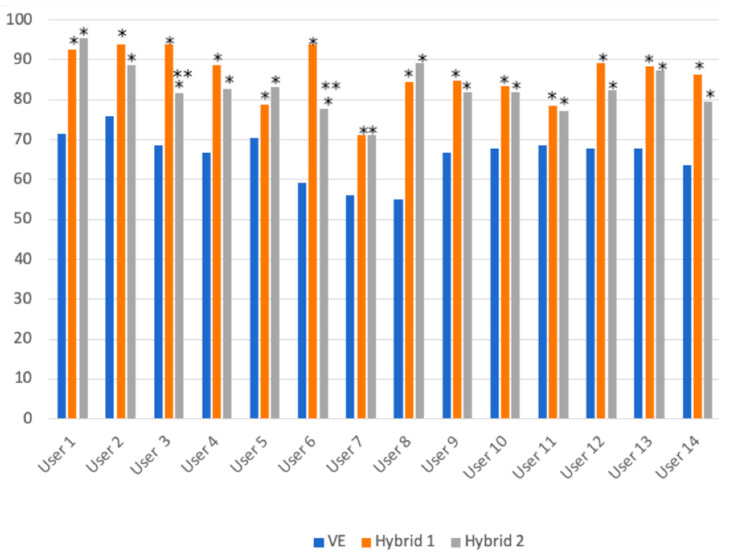
The accuracy to predict an ischemic fractional flow reserve based on each operator’s visual estimation, Hybrid 1 or Hybrid 2 approaches. * above each column depicts a *p*-value < 0.05 between VE and Hybrid approach; ** a *p* < 0.005 between the Hybrid 1 and Hybrid 2 approach.

When comparing the operator decision for revascularization based on VE with measured FFR as gold standard, there was an average accuracy of 66.1 ± 5.8%, ranging from 55% to 75.8% for different operators (Table 4, Figure 1). 

The overall agreement for the indication of revascularization based only on the VE of stenoses was moderate (k = 0.448, *p* < 0.0001). Based on our analysis we did not prove a significant correlation between the operator’s experience quantified by the years of interventional cardiology practice (r = –0.48; *p* = 0.08) or annual PCI load (r = –0.44; *p* = 0.11) and the accuracy in predicting the functional significance of the stenoses. When the decision for revascularization was based on votes of the majority (at least eight), it led to a slightly better accuracy, 67.7%. When considering the majority opinion of the top three the accuracy was 75.9%, but the difference was not significant statistically (*p* = 0.09), and there was a better agreement between these two group decisions (Cohen kappa 0.773). Still, at the individual level there was only a moderate inter-observer agreement, even between the top three raters, concerning the indication for revascularization with Cohen kappa values between 0.55–0.68 (Table 5).

When classifying the lesions based on the decision of the majority, we observed that in the category <50% there were only three lesions with ischemic FFR (12%), while for 50–70% stenoses only 28.3% were functionally significant and in the category >70% stenoses, 70.8% were functionally significant (Table A2). The average accuracy for the operators’ decision for revascularization in each of these groups were 81.2%, 59.1%, 65.6%, respectively. However, the accuracy improved when we used the decision of the top three operators (88%, 75% and 70.8%), especially for the intermediate degree stenoses (Table A2). Evaluating non-intermediate lesions (<50% or >70%) in terms of predicting the need for revascularization, between top three and the overall majority, we obtained an almost perfect agreement, with a Cohen kappa 0.914 (*p* < 0.0001), while the agreement for the intermediate lesions was only fair with a Cohen kappa 0.385 (*p* = 0.001).

The Hybrid 1 approach showed the best average accuracy, 86.3 ± 6.7% (*p* < 0.0001 for Hybrid 1 vs. VE comparison), while for the Hybrid 2 approach the accuracy was 82.9 ± 6% (*p* < 0.0001 for Hybrid 2 vs. VE comparison). On average there were 14.6 more invasive functional assessments (ranging from −6 to +48) using the Hybrid 1 approach compared to the Hybrid 2 approach (Table A5). However, the difference between Hybrid 1 and Hybrid 2 approaches was not statistically significant in terms of accuracy (*p* = 0.166). Furthermore, the overall agreement for revascularization significantly improved for both hybrid approaches (Cohen k value of 0.717, *p* < 0.0001 for Hybrid 1 and Cohen k = 0.676, for Hybrid 2, *p* < 0.0001).

Table A3 displays the results of the Hybrid 1 strategy for each category of lesion severity (using a 3-grade scale), as judged by each operator. The average (range) accuracies were 87.1% (75–100%), 94.3% (80–100%) and 79.5% (61.4–95.1%) for lesions evaluated as <50%, 50–70% and >70% respectively. There were significant differences between these values (<50% vs. 50–70%, *p* = 0.008; 50–70% vs. >70, *p* < 0.0001; <50 vs. >70, *p* = 0.044).

**Figure 2:**
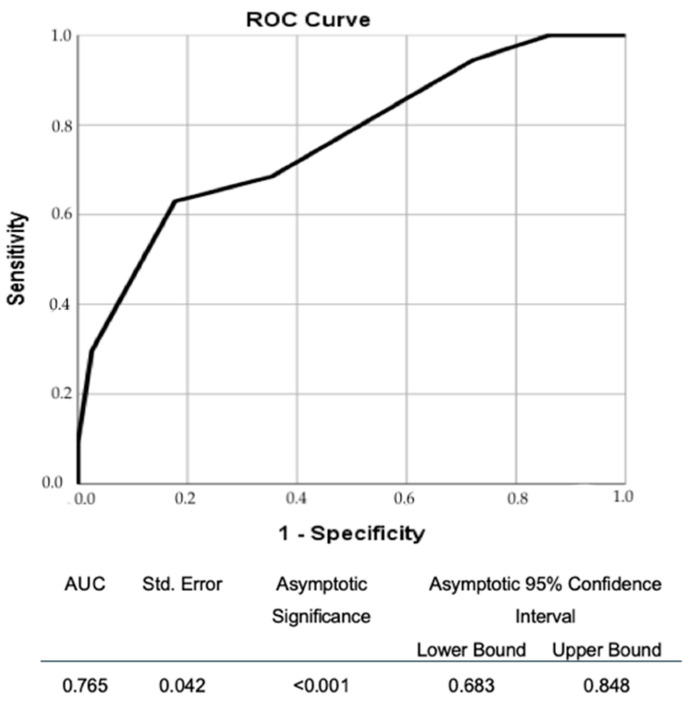
Receiver operator characteristic curve for the relation between diameter stenosis severity (based on 7-degree classification according to the decision of the majority) and the functional significance (FFR ≤ 0.8).

We performed the ROC analysis for the dependence between the DS severity (as quantified by each operator and by the decision of majority) and the functional significance of the lesions (Figure 2, Table A6). The values for the area under the curve for different operators were similar (range 0.706–0.802), without a significant difference between the largest and the lowest value, while the value for AUC obtained using the decision of the majority lied also in this range (0.752).

## 4. Discussions

### 4.1. VE of the DS

The first step in the evaluation of a coronary lesion is the estimation of the maximum degree of diameter reduction, at the level of the stenosis, compared to the reference diameter. The reference is considered the average diameter of the proximal and distal segments. There are several reports on inter- and intra-observer variability in DS reporting [14,15] performed several decades earlier. We asked the operators to report the range of DS, which allowed us to perform a further classification in more broader severity groups, as recommended by current guidelines. The process of grading resulted in a Kendall’s W value of 0.866, (*p* < 0.005) for the 7-grade severity scale, considered to illustrate an overall good agreement, and a Kendall’s W value of 0.748, (*p* < 0.005) for the 3-grade severity scale (a value of 1 means a perfect agreement). This overall good agreement measured with Kendall’s W should be interpreted cautiously as there were large variations between evaluators regarding number of lesions classified in each interval of the 3-grade severity scale (Table A2 and Table A3). Because of this wide inter-observer variation in grading the lesions, we used the decision of the majority in order to classify the lesions for some.

### 4.2. Need for Invasive Functional Evaluation

In our study we asked the operators to indicate the need for invasive functional evaluation to assess the lesions in an ideal scenario without any constraints, for guiding the therapeutic decision regarding revascularization. There were large differences between the operator’s opinions in considering the need for functional evaluation with fair agreement (Fleiss kappa of 0.214), whereas between the top three operators there was a moderate agreement with an average Cohen kappa of 0.534 (Table 2). The evaluation was considered necessary most often when the lesion was classified as intermediate (DS between 50–70%), as generally recommended, without a significant influence based on the vessel involved or the proximal location of the lesion. However, at individual level there were wide variations even when we quantified the need for evaluation based on the 3-grade stenosis classification. These wide differences in opinion may reflect differences in experience using functional evaluation or other lesion characteristics impacting the operator’s opinion. There are registry data supporting the significant variation in utilization of FFR during every day practice in patients with intermediate degree stenosis [10]. To overcome the variability introduced by the operator’s subjectivity, it might be indicated to use group decision. Analyzing the decision of the majority (at least eight votes) and the decision of the top three operators (at least two votes), we obtained a much better agreement with a Cohen k of 0.729 (Table 2). This finding may support the use of group decision when evaluating the need for invasive functional assessment.

### 4.3. The Functional Significance Prediction by VE

In our cohort of patients there were 54 (40.6%) lesions with an ischemic FFR value. Considering the three grade DS categories (<50%, 50–70% and >70 as classified by the majority of the operators), there were 3, 17 and 34 lesions, representing 12%, 28.3% and 70.8% from each category. Our data compare well to those derived from the FAME trial where in lesions >50% DS by VE [16] there were 35% significant lesions in the intermediate degree stenosis (50–70%), while among those >70% DS there were 80% functionally significant lesions. It means that for visually intermediate lesions and for those >70% (non-sub-totally occluded lesions) there are around one third and three fourths of significant lesions respectively. These findings argue against using the generally proposed 50% threshold for defining the significance of the lesions. In our study the average accuracy of identifying functional significant lesions in the intermediate severity range was 59% ranging from 29.8% to 74.6%, meaning that for some evaluators the accuracy was less than 50% (worse than “tossing a coin”). Moreover, it decreased slightly to 56.7% when we modelled the decision based on the opinion of the majority of the operators (we used the threshold of at least 8 positive votes). However, it improved significantly when we used the group decision of the top three operators (to 75.9%). In fact, only in the intermediate degree stenoses by VE the discriminating capacity of top three operators performed better compared to the overall accuracy (Table A2). It is worth mentioning here that in our study the evaluators’ accuracy did not correlate with his/her overall previous experience in the field of interventional cardiology, as evaluated by means of years of experience or the annual PCI load. There are other reports like Lopez et al. [17] showing similar accuracy in functional significance prediction based on consensus VE evaluation for experienced operators evaluating intermediate DS lesions as demonstrated by QCA, while the reclassification of revascularization adequacy after FFR reached almost 50% in the French registry R3F [18]. It is known that fore severe lesions DS might be overestimated when compared to QCA [13]. However, VE was proven to be a better clinical tool compared to QCA for evaluating the functional significance [13]. Also, Shah et al. [19] demonstrated that among lesions considered as non-significant on QCA, those evaluated visually as significant had a higher long-term risk compared to non-significant lesions based on both VE and QCA.

We also evaluated the dependence between the VE of degree of DS and the functional significance of the stenoses. We performed the ROC analysis for each grader (Figure 2, Table A6). Overall, there were similar results as measured by the AUC. The difference between the largest (Operator 9) and the smallest values (Operator 3) was not significantly different (*p* = 0.116; AUC difference −0.097; 95% CI −0.217–0.029). We may conclude that we had similar capabilities for discrimination based on DS evaluation by VE for all operators, which may support, alongside with the general overall agreement between the evaluators in grading the stenosis (as illustrated by the Kendall w of 0.866 for the agreement on the 7-grade severity scale), the validity of our methodology of lesion evaluation. When analyzing the curve obtained using the classification based on the decision of the majority (less prone to individual observer variation), we may identify the threshold values for DS (evaluated by VE) which may guide the strategy of functional evaluation (Figure 2). Using a threshold of <50% for ruling out significant stenosis we obtain a sensitivity of 94.4%, while using a value of >80% to define functional significance we obtain a specificity of 97.5%. If we decrease the threshold to 70% the specificity decreases to 77.2% which seems not appropriate. We must also acknowledge the limitation of VE in discriminating the DS severity around the 70% threshold because of the tendency of overestimation by VE compared to QCA [13]. Classifying a lesion as 60–70% will support invasive functional assessment, while grading it as 70–80% might preclude it, making the intermediate-high grade lesions less prone to be evaluated functionally.

This supports the use of a >80% DS by VE to define functional significance, as was mentioned in a recent consensus [20], a less restrictive strategy than the one recommended in the European Guidelines for Revascularization [21] which advocates a threshold of 90% to justify revascularization based solely on angiographic evaluation of stenosis severity. It is important to mention that in every-day practice we may have additional clinical information, which should always be used to support the decision to revascularization, when the DS does not meet those specific criteria. A further evaluation of the subjective decision (both DS quantification and functional significance prediction) performed specifically for these intermediate-high DS lesions should be envisaged.

The Hybrid 1 scenario attempted to simulate an ideal every-day practice. The operator performs the angiography, evaluates it visually and decides if the vessel requires further functional evaluation to define the need for revascularization. Then, if the VE is considered to be enough, no further information will be obtained and the lesion will be treated according to the VE quantification, otherwise, functional evaluation is performed to inform the decision. Using this approach, which incorporates the previous experience of the operator, an overall accuracy of 86.3% (71.2–93.9%) was obtained, which is significantly higher compared to the VE accuracy (*p* < 0.0001). The scenario of performing functional evaluation only in intermediate DS (Hybrid 2), as classified by each observer, resulted in a nonsignificant lower accuracy compared to the Hybrid 1 scenario (82.9 vs 86.3%; *p* = 0.166). There were on average 14.6 less functional evaluations with the Hybrid 2 strategy compared to the Hybrid 1 strategy.

This significant increase in accuracy for the Hybrid 1 strategy compared to VE originated from a better classification of intermediate DS lesions, which were the most frequently indicated as needing functional evaluation (see Table A4). Consequently, in intermediate DS lesions the overall accuracy of the Hybrid 1 strategy reached 94.3% (Table A3). Our data does not support the routine use of a Hybrid 1 like strategy (invasive functional assessment in all lesions deemed necessary by the operator) over a Hybrid 2 like strategy (restricting functional assessment only to intermediate DS lesions, as evaluated visually), as this does not result in a significant increase in accuracy at the expense of increasing the need for invasive functional evaluation. Furthermore, using the Hybrid 1 strategy there is a worse classification in terms of functional significance for those lesions graded as >70% compared to <50% (79.5% vs. 86.9%, *p* = 0.044), most probably related to the tendency of overestimating the significance of tight lesions by VE. It is intriguing however that even for a hypothetically liberal use of invasive functional testing the overall accuracy is limited to almost 85% (with a maximum of 93.9%), while the best individual accuracy was achieved using a Hybrid 2 scenario (95.5%).

When a lesion is classified as <50% it means that it is regarded as probably non-significant, and a functional assessment might be considered overall in only one fifth of the cases, or in less than one tenth based on the decision of the majority. By visual estimation of the functional significance there is a correct decision in 80–90% of these cases. When a lesion is classified in the >70% range it means that it is regarded as probably significant, and a functional assessment might be considered overall in only 20–30% of the cases. By visual estimation of the functional significance there is a correct decision in 60–70% of the cases. Only for intermediate degree stenosis there is a high level of indication for invasive functional testing, while the chance of a correct prediction of functional significance is unacceptably low (50–60%). These are the reasons for which we interpreted the 3-grade classification also as a nominal variable when we analyzed the inter-observer agreement in DS estimation, and we demonstrated that there is only a fair overall agreement (k = 0.39 ± 0.07) and a moderate agreement even between the top three operators. This means that there are in fact important operator dependent variations in terms of clinical decision. It seems that, bearing in mind the limitations of our study, there is still some room for improvement in the way we currently integrate the use of invasive functional testing with the information obtained by visual evaluation of coronary angiographies.

### 4.4. Limitations

We assumed that the data that were made available were based entirely on the operator’s opinion after proper evaluation of the included lesions. We could not control for evaluator’s accidental errors during data collection in the central database. The images used (two views for each coronary artery) were selected as being those which display the highest DS based on the author’s opinion. The operators annotated these images and did not have access to the entire coronary evaluation as is the case in usual practice. Moreover, a web-based annotation tool was employed for evaluating the medical images. Real life accuracy may be better due to the integration of the overall clinical assessment of the patients, which is based not only on angiography images. We did not perform an analysis of intra-observer variability which would have been of much interest especially for the top three raters. For the purposes of our study we have considered FFR as golden standard for defining the functional significance of the stenoses as currently, without taking into consideration its possible limitation. Fractional flow rate reflects the hemodynamic severity of coronary stenosis in the proximal artery assuming that maximum hyperemia may be achieved; abnormal resistance in the microcirculation may lead to a decreased maximum coronary flow and subsequently to a smaller pressure gradient across the stenosis [22]; to the best of our knowledge the clinical impact of these FFR limits is unknown.

## 5. Conclusions

Visual evaluation of coronary lesions is an important tool for evaluation during ICA. There are significant inter-observer differences in reporting the degree of DS. The accuracy of VE for predicting the functional significance is largely dependent on the operator, and the worst performance is seen in the evaluation of intermediate degree stenoses. The average accuracy of the visual prediction of functional significance of intermediate degree stenosis was 55%, advocating against the sole use of VE to support the decision of revascularization. The whole-group decision did not improve the accuracy of functional significance prediction in intermediate lesions. The visual prediction of functional significance for intermediate stenosis may increase up to 75% in accuracy for some operators, most probably based on specific personal experience. There were wide variations between the evaluators regarding the need for invasive functional assessment. Intermediate degree stenoses are most likely to be evaluated functionally. A more liberal use of functional evaluation (based on physician decision), compared to the one considering only intermediate degree lesions only, does not result in an improvement of the overall accuracy, and has the disadvantage of an increase in number of functional evaluations.

## Figures and Tables

**Table 1:** The general characteristics of the cohort (*n* = 86 subjects) and lesions (*n* = 133) evaluated.

Age (years)	62.6 ± 8.9
Sex (male) *n* (%)	58 (67.4%)
Smoking *n* (%)	44 (51.2%)
Arterial hypertension *n* (%)	76 (88.3%)
Diabetes mellitus *n* (%)	26 (30.2%)
Dyslipidemia *n* (%)	73 (83.7%)
History of acute coronary syndrome *n* (%)	36 (41.8%)
History of revascularization, *n* (%)	46 (53.5%)
Left ventricular ejection fraction (%)	50.2 ± 6.6%
Treatment Medical follow-up, *n* (%) PCI, *n* (%) CABG, *n* (%)	38 (44.2%) 44 (51.2%) 4 (4.6%)
Artery LAD, *n* (%) LCX, *n* (%) RCA, *n* (%)	71 (53.4%) 30 (22.5%) 32 (24.1%)
FFR	
Significant, *n* (%)	54 (40.6%)
Value mean (min–max)	0.67 (0.19–0.8)
Non-significant, *n* (%)	79 (59.4%)
Value mean (min–max)	0.88 (0.81–0.98)
LAD left anterior descending artery; LCx left circumflex artery; RCA right coronary artery	

**Table 2:** The agreement for the requirement of invasive evaluation.

	Cohen Kappa	*p*-Value
Top 3 Operators comparisons		
Operator 1 vs. Operator 2	0.545	<0.001
Operator 1 vs. Operator 5	0.51	<0.001
Operator 2 vs. Operator 5	0.547	<0.001
Majority of Top 3 vs. overall majority		
	0.729	<0.001

**Table 3:** Correlations for the decision to perform functional invasive assessment.

	Vessel		Proximal vs. Distal		Intermediate vs. Non-Intermediate	
	Phi	*p*-Value	Phi	*p*-Value	Phi	*p*-Value
Operator 1	0.115	0.417	0.011	0.898	0.607	<0.001
Operator 2	0.209	0.056	0.039	0.65	0.478	<0.001
Operator 3	0.07	0.724	0.055	0.532	0.204	0.02
Operator 4	0.179	0.119	−0.016	0.854	0.626	<0.001
Operator 5	0.172	0.142	0.062	0.476	0.441	<0.001
Operator 6	0.162	0.181	0.078	0.377	−0.065	0.459
Operator 7	0.293	0.03	−0.126	0.146	−0.092	0.292
Operator 8	0.095	0.559	0.144	0.262	0.078	0.378
Operator 9	0.07	0.722	−0.078	0.37	0.468	<0.001
Operator 10	0.045	0.872	−0.03	0.725	0.69	<0.001
Operator 11	0.043	0.885	−0.123	0.159	0.479	<0.001
Operator 12	0.124	0.363	0.068	0.738	0.618	<0.001
Operator 13	0.045	0.876	0.04	0.651	0.763	<0.001
Operator 14	0.119	0.654	0.173	0.184	0.628	<0.001

**Table 4:** The statistics for each individual operator decision, majority vote, top three operators and the hybrid approach concerning revascularization versus the golden-standard fractional-flow rate.

Operator	Sensitivity	Specificity	PPV	NPV	Accuracy
Operator 1					
Visual	81.5%	64.6%	61.1%	83.6%	71.4%
Hybrid_1	94.4%	91.1%	87.9%	96.0%	92.5%
Hybrid_2	94.4%	96.2%	94.4%	96.2%	95.5%
Operator 2					
Visual	74.1%	76.9%	69.0%	81.1%	75.8%
Hybrid_1	96.3%	92.3%	89.7%	97.3%	93.9%
Hybrid_2	81.5%	92.4%	88.0%	88.0%	88.6%
Operator 3					
Visual	66.7%	70.1%	61.0%	75.0%	68.7%
Hybrid_1	88.9%	97.4%	96.0%	92.6%	93.9%
Hybrid_2	77.8%	82.3%	75.0%	84.4%	81.7%
Operator 4					
Visual	42.6%	83.3%	63.9%	67.7%	66.7%
Hybrid_1	75.9%	97.4%	95.3%	85.4%	88.6%
Hybrid_2	70.4%	89.9%	82.6%	81.6%	82.6%
Operator 5					
Visual	63.0%	75.6%	64.2%	74.7%	70.5%
Hybrid_1	81.5%	76.9%	71.0%	85.7%	78.8%
Hybrid_2	79.6%	84.8%	78.2%	85.9%	83.3%
Operator 6					
Visual	66.7%	53.9%	50.7%	69.5%	59.2%
Hybrid_1	100.0%	89.5%	87.1%	100.0%	93.8%
Hybrid_2	88.9%	67.1%	64.9%	89.8%	77.7%
Operator 7					
Visual	81.5%	38.5%	47.8%	75.0%	56.1%
Hybrid_1	96.3%	53.8%	59.1%	95.5%	71.2%
Hybrid_2	96.3%	53.2%	58.4%	95.5%	71.2%
Operator 8					
Visual	92.6%	28.0%	48.1%	84.0%	55.0%
Hybrid_1	100.0%	73.3%	73.0%	100.0%	84.5%
Hybrid_2	96.3%	79.7%	76.5%	96.9%	89.1%
Operator 9					
Visual	75.9%	60.3%	56.9%	78.3%	66.7%
Hybdrid_1	92.6%	79.5%	75.8%	93.9%	84.8%
Hybrid_2	88.9%	75.9%	71.6%	90.9%	81.8%
Operator 10					
Visual	66.7%	68.4%	59.0%	75.0%	67.7%
Hybdrid_1	92.6%	77.2%	73.5%	93.8%	83.5%
Hybrid_2	90.7%	75.9%	72.1%	92.3%	82.0%
Operator 11					
Visual	67.9%	69.2%	60.0%	76.1%	68.7%
Hybrid_1	86.8%	73.1%	68.7%	89.1%	78.6%
Hybrid_2	83.3%	70.9%	66.2%	86.2%	77.1%
Operator 12					
Visual	55.6%	76.6%	62.5%	71.1%	67.9%
Hybdrid_1	90.7%	88.3%	84.5%	93.2%	89.3%
Hybrid_2	83.3%	79.7%	73.8%	87.5%	82.4%
Operator 13					
Visual	56.6%	75.6%	61.2%	72.0%	67.9%
Hybrid_1	83.0%	92.3%	88.0%	88.9%	88.5%
Hybrid_2	83.3%	88.6%	83.3%	88.6%	87.8%
Operator 14					
Visual	61.1%	65.4%	55.0%	70.8%	63.6%
Hybrid_1	88.9%	84.6%	80.0%	91.7%	86.4%
Hybrid_2	83.3%	75.9%	70.3%	87.0%	79.5%
Average					
Visual	68%	64.7%	58.6%	75.3%	66.1%
Hybrid_1	90.6%	83.3%	80.7%	93.1%	86.3%
Hybrid_2	856%	79.5%	75.4%	89.3%	82.9%
Majority vote	64.8%	69.6%	59.3%	74.3%	67.7%
Top 3 operators	77.8%	74.7%	67.7%	83.1%	75.9%

NPV negative predictive value; PPV positive predictive value.

**Table 5:** The agreement for need of revascularization.

	Cohen Kappa	*p*-Value
Operator 1–Operator 2	0.57	<0.001
Operator 1–Operator 5	0.68	<0.001
Operator 2–Operator 5	0.55	<0.001
Majority of Top 3 vs. overall majority		
	0.773	<0.001

## Data Availability

Upon request from the corresponding author.

## Appendix A

**Table A1:** Stenosis severity as graded by each operator and based on decision of majority.

	<30%		30–50%		50–60%		60–70%		70–80%		80–90%		>90%	
	*n*	%	*n*	%	*n*	%	*n*	%	*n*	%	*n*	%	*n*	%
Operator 1	11	8.3%	16	12.%	55	41.4%	35	26.3%	9	6.8%	4	3.0%	3	2.3%
Operator 2	10	7.6%	30	22.7%	33	25.00%	30	22.7%	18	13.6%	6	4.6%	5	3.8%
Operator 3	13	9.9%	32	24.4%	29	22.1%	16	12.2%	24	18.3%	9	6.9%	8	6.1%
Operator 4	25	18.9%	32	24.2%	25	18.9%	16	12.1%	14	10.6%	13	9.9%	7	5.3%
Operator 5	24	18.2%	33	25.0%	21	15.9%	14	10.6%	26	19.7%	8	6.1%	6	4.6%
Operator 6	5	3.9%	8	6.2%	22	16.9%	31	23.9%	31	23.9%	24	18.5%	9	6.9%
Operator 7	2	1.5%	2	1.5%	16	12.1%	29	22%	58	43.9%	16	12.1%	9	6.8%
Operator 8	1	0.8%	21	16.3%	27	20.9%	30	23%	28	21.7%	20	15.5%	2	1.6%
Operator 9	3	2.3%	22	16.7%	23	17.4%	24	18.2%	38	28.78%	18	13.6%	4	3.0%
Operator 10	10	7.5%	15	11.3%	22	16.5%	31	23.3%	25	18.8%	23	17.3%	7	5.3%
Operator 11	15	11.5%	17	13%	12	9.2%	17	13%	23	17.6%	29	22.1%	18	13.4%
Operator 12	2	1.5%	11	8.4%	33	25.2%	24	18.3%	28	21.4%	21	16.%	12	9.2%
Operator 13	17	13%	23	17.6%	30	22.9%	22	16.8%	24	18.3%	9	6.8%	6	4.6%
Operator 14	12	9.1%	22	16.7%	29	22%	18	13.6%	23	17.4%	18	13.6%	10	7.6%
Average	10.7	8.1%	20.3	15.4%	26.9	20.5%	24.1	18.3%	26.4	20.1%	15.6	11.9%	7.6	5.8%
Majority	11	8.3%	14	10.5%	43	32.3%	17	12.8%	30	22.6%	13	9.8%	5	3.8%

**Table A2:** Accuracy in predicting functional significance (FFR ≤ 0.8) based on visual estimation (VE) and stenosis severity.

	<50%			50–70%			>70%			Total		
	Total	VE Correct	%	Total	VE Correct	%	Total	VE Correct	%	Total	VE Correct	%
Operator 1	27	24	88.9%	90	58	64.4%	16	13	81.3%	133	95	71.4%
Operator 2	40	33	82.5%	63	47	74.6%	29	20	69.0%	132	100	75.8%
Operator 3	45	34	75.6%	45	30	66.7%	41	26	63.4%	131	90	68.7%
Operator 4	57	44	77.2%	41	21	51.2%	34	23	67.6%	132	88	66.7%
Operator 5	57	46	80.7%	35	19	54.3%	40	28	70.0%	132	93	70.5%
Operator 6	13	11	84.6%	53	32	60.4%	64	34	53.1%	130	77	59.2%
Operator 7	4	3	75.0%	45	26	57.8%	83	45	54.2%	132	74	56.1%
Operator 8	22	20	90.9%	57	17	29.8%	50	34	68.0%	129	71	55.0%
Operator 9	25	21	84.0%	47	28	59.6%	60	39	65.0%	132	88	66.7%
Operator 10	25	20	80.0%	53	34	64.2%	55	36	65.5%	133	90	67.7%
Operator 11	32	27	84.4%	29	19	65.5%	70	44	62.9%	131	90	68.7%
Operator 12	13	10	76.9%	57	40	70.2%	61	39	63.9%	131	89	67.9%
Operator 13	40	33	82.5%	52	27	51.9%	39	29	74.4%	131	89	67.9%
Operator 14	34	25	73.5%	47	27	57.4%	51	32	62.7%	132	84	63.6%
			81.2%			59.1%			65.8%			66.1%
Majority *	25	22	88.0%	60	45	56.7%	48	34	70.8%	133	101	75.9%
Top 3 *	25	22	88.0%	60	34	75.0%	48	34	70.8%	133	90	67.7%

* classification based on overall agreement for the degree of severity.

**Table A3:** Performance of the Hybrid 1 (Hy1) strategy based on three degrees of stenosis (as judged by each operator).

	<50%			50–70%			>70%			Total		
	Total	Hy 1 Correct (*n*)	%	Total	Hy 1 Correct (*n*)	%	Total	Hy 1 Correct (*n*)	%	Total	Hy 1 Correct (*n*)	%
Operator 1	27	24	88.9%	90	85	94.4%	16	14	87.5%	133	123	92.5%
Operator 2	40	38	95.0%	63	60	95.2%	29	26	89.7%	132	124	93.9%
Operator 3	45	40	88.9%	45	44	97.8%	41	39	95.1%	131	123	93.9%
Operator 4	57	44	77.2%	41	41	100.0%	34	32	94.1%	132	117	88.6%
Operator 5	57	48	84.2%	35	28	80.0%	40	28	70.0%	132	104	78.8%
Operator 6	13	13	100.0%	53	53	100.0%	64	56	87.5%	130	122	93.8%
Operator 7	4	3	75.0%	45	40	88.9%	83	51	61.4%	132	94	71.2%
Operator 8	22	22	100.0%	57	52	91.2%	50	35	70.0%	129	109	84.5%
Operator 9	25	22	88.0%	47	43	91.5%	60	47	78.3%	132	112	84.8%
Operator 10	25	21	84.0%	53	52	98.1%	55	38	69.1%	133	111	83.5%
Operator 11	32	29	90.6%	29	27	93.1%	70	47	67.1%	131	103	78.6%
Operator 12	13	10	76.9%	57	55	96.5%	61	52	85.2%	131	117	89.3%
Operator 13	40	34	85.0%	52	50	96.2%	39	32	82.1%	131	116	88.5%
Operator 14	34	29	85.3%	47	46	97.9%	51	39	76.5%	132	114	86.4%
Average			86.9%			94.3%			79.5%			86.3%

**Table A4:** Need for functional evaluation for each operator based on stenosis severity (as graded by each operator).

	<50%			50–70%			>70%				Total	
	Total *n*	Need for Functional Evaluation		Total *n*	Need for Functional Evaluation		Total *n*	Need for Functional Evaluation		Total *n*	Need for Functional Evaluation	
		*n*	%		*n*	%		*n*	%		*n*	%
Operator 1	27	0	0.0%	90	70	77.8%	16	1	6.3%	133	71	53.4%
Operator 2	40	17	42.5%	63	54	85.7%	29	10	34.5%	132	81	61.4%
Operator 3	45	24	53.3%	45	43	95.6%	41	27	65.9%	131	94	71.8%
Operator 4	57	0	0.0%	41	38	92.7%	34	23	67.7%	132	61	46.2%
Operator 5	57	15	26.3%	35	21	60.0%	40	0	0.0%	132	36	27.3%
Operator 6	13	10	76.9%	53	53	100.0%	64	34	53.1%	130	97	74.6%
Operator 7	4	0	0.0%	45	32	71.1%	83	8	9.6%	132	40	30.3%
Operator 8	22	10	45.5%	57	49	86.0%	50	2	4.0%	129	61	47.3%
Operator 9	25	2	8.0%	47	36	76.6%	60	22	36.7%	132	60	45.5%
Operator 10	25	1	4.0%	53	44	83.0%	55	10	18.2%	133	55	41.4%
Operator 11	32	8	25.0%	29	22	75.9%	70	14	20.0%	131	44	33.6%
Operator 12	13	0	0.0%	57	55	96.5%	61	27	44.3%	131	82	62.6%
Operator 13	40	1	2.5%	52	45	86.5%	39	7	18.0%	131	53	40.5%
Operator 14	34	12	35.3%	47	45	95.7%	51	14	27.5%	132	71	53.8%
Average		7.1	22.8%		43.4	84.5%		14.2	29.0%		64.7	49.2%
Majority *	25	1	4.0%	60	53	88.3%	48	11	22.9%	133	65	48.9%
Top 3 *	25	2	8.0%	60	48	80.0%	48	15	31.3%	133	65	48.9%

* classification based on overall agreement for the degree of severity.

**Table A5:** The number of fractional-flow rate (FFR) measurements in the two hybrid approaches and the difference between these concerning the number of FFR.

	Hybdrid 1	Hybrid 2	Hybrid 1-Hybrid 2
Operator 1	71	77	−6
Operator 2	81	63	18
Operator 3	94	46	48
Operator 4	61	41	20
Operator 5	36	35	1
Operator 6	97	53	44
Operator 7	40	45	−5
Operator 8	61	57	4
Operator 9	60	47	13
Operator 10	55	53	2
Operator 11	44	29	15
Operator 12	82	57	25
Operator 13	53	52	1
Operator 14	71	47	24
Average	64.7	50.1	14.6

**Table A6:** Results from area under ROC curve analysis for the relation between degree of DS and the functional significance of the lesions (FFR ≤ 0.8).

				Asymptotic 95% Confidence Interval	
DS Classification Method	AUC	Standard Error	Asymptotic Significance	Lower Bound	Upper Bound
Decision of majority	0.753	0.042	0.000	0.670	0.836
Operator 1	0.741	0.043	0.000	0.657	0.825
Operator 2	0.762	0.043	0.000	0.678	0.847
Operator 3	0.706	0.047	0.000	0.614	0.797
Operator 4	0.732	0.045	0.000	0.643	0.821
Operator 5	0.769	0.042	0.000	0.687	0.851
Operator 6	0.736	0.044	0.000	0.649	0.822
Operator 7	0.716	0.047	0.000	0.624	0.808
Operator 8	0.759	0.043	0.000	0.674	0.843
Operator 9	0.802	0.040	0.000	0.723	0.881
Operator 10	0.762	0.043	0.000	0.678	0.846
Operator 11	0.760	0.044	0.000	0.675	0.846
Operator 12	0.740	0.045	0.000	0.651	0.829
Operator 13	0.744	0.045	0.000	0.656	0.832
Operator 14	0.724	0.046	0.000	0.634	0.815

AUC area under the curve; DS diameter stenosis; FFR fractional flow reserve; ROC receiver operator characteristic.
